# The effect of obesity on the GnRH stimulation test in girls with idiopathic central precocious puberty

**DOI:** 10.1007/s00431-025-06077-w

**Published:** 2025-03-17

**Authors:** Ulku Gul Siraz, Ayse Karadag, Nazlı Sultan Ozsoy, Emine Kaygi Tartici, Aynura Aliyeva, Selim Kurtoglu, Nihal Hatipoglu

**Affiliations:** Department of Pediatric Endocrinology, Faculty of Medicine, Erciyes University, Kayseri, Turkey

**Keywords:** Idiopathic central precocious puberty, Thelarche, Gonadotropin releasing hormone stimulation test, Obesity

## Abstract

**Abstract:**

The gonadotropin-releasing hormone (GnRH) stimulation test is essential for diagnosing idiopathic central precocious puberty (ICPP). Research provided that luteinizing hormone (LH) levels during the test are lower in overweight and obese girls. This study aims to establish diagnostic cut-off values in the GnRH stimulation test specifically for overweight and obese girls with ICPP. Retrospective data from 925 girls diagnosed with ICPP or premature thelarche (PT) who underwent GnRH testing were analyzed. Patients were categorized into normal weight (NW) and overweight/obese (OW) groups based on Body Mass Index Standard Deviation Score (BMI-SDS), with BMI-SDS ≥ 1 indicating OW. Only patients with Tanner stage 2 or 3 breast development were included. The mean age at diagnosis was 7.9 ± 1.1 years in ICPP and 6.4 ± 1.4 years in PT. Among the patients, 455 (49.2%) were OW. In the OW-ICPP group, the peak LH cut-off was 3.56 IU/L (AUC:0.733; sensitivity:69.2%, specificity:64%), and the peak LH/FSH ratio was 0.29 (AUC:0.828; sensitivity:77.1%, specificity:76.3%). For NW patients, the peak LH cut-off was 4.75 IU/L (AUC:0.809; sensitivity:77.1%, specificity:70.7%), and the peak LH/FSH ratio was 0.3 (AUC:0.926; sensitivity: 86.3%, specificity: 86%). In the peak LH cut-off model, the multivariate analysis identified BMI-SDS as a significant negative predictor (OR:0.585, 95%CI: 0.477–0.717, *p* < 0.001), showing a strong inverse relationship. Similarly, in the peak LH/FSH ratio model, BMI-SDS remained a significant negative predictor (OR: 0.744, 95% CI: 0.614–0.902, *p* < 0.001).

**Conclusion:**

In this study, gonadotropin responses during the GnRH stimulation test were lower in overweight and obese girls with Tanner stage 2 and 3 ICPP compared to standard thresholds. It is important to utilize the GnRH test alongside clinical findings when diagnosing these patients, as responses below standard values do not rule out precocious puberty. This highlights the need for tailored diagnostic criteria to ensure timely and accurate diagnosis in this population.

**Table:** 

**What is known:** *• Obesity is a risk factor for early puberty.*
**What is new:** *• In girls with idiopathic central precocious puberty, obesity leads to lower values in the GnRH stimulation test compared to normal ranges. This may result in missed diagnoses, emphasizing the need to evaluate cases thoroughly with clinical data.*

## Introduction

The age of puberty onset is accepted as eight years in girls and nine years in boys, and its onset before this age is considered precocious puberty. Central precocious puberty is more common in girls than in boys and is frequently idiopathic. Additionaly, idiopathic central puberty precocity (ICPP) is observed more frequently in overweight and obese girls compared to normal-weight girls [1, 2].

Although a basal LH value of 0.3 IU/L and above is considered high, basal FSH and LH values do not provide sufficient guidance in diagnosing ICPP [3, 4]. It has been reported that a basal LH value > 1.0 IU/L has a positive predictive value of 96.4% [5].

Examination findings and basal hormonal evaluation may not always be definitive for diagnosing CPP. The Gonadotropin-Releasing Hormone Stimulation Test (GnRH test) is the gold standard for differentiating CPP from other causes of early puberty [6, 7]. An increase in LH level above 5 IU/L and a peak LH/FSH ratio greater than 0.66 with GnRH stimulation indicates that the hypothalamus-pituitary-gonad axis is active [6–10].

Obesity is a potent inducer of puberty, with increased leptin levels at the pre-pubertal period and increased bone age due to aromatization. Leptin signaling stimulates GnRH by binding to leptin receptors and activating kisspeptin [2, 11, 12]. In addition, insulin resistance increases serum ovarian and adrenal-derived androgens and decreases sex hormone-binding globulin (SHBG) levels [13, 14]. However, it should be considered that peak LH response may be lower in obese children due to the suppressive effect of androgens and estrogens on LH [15, 16].

Additionally, obesity in pre-pubertal and early pubertal girls is associated with nocturnal changes and decreased LH secretion compared with normal-weight girls. On the other hand, it has been associated with an increased frequency of LH secretion in late puberty [17].

In this retrospective study, we aimed to determine the effect of obesity on GnRH stimulation test, in cases with thelarche stage 2 and 3, and to investigate whether the cut-off values of peak LH values and peak LH/FSH ratios in diagnosing idiopathic central precocious puberty in overweight and obese girls compared to normal-weight girls.

## Sample and method

Retrospective data was collected from 925 girls who underwent GnRH testing between January 2012 and December 2023, diagnosed with ICPP and PT, characterized by an onset of breast development before age 8, at Erciyes University, Faculty of Medicine, Department of Paediatric Endocrinology. The decimal age at the time of the GnRH test was recorded.

The PT group consisted of patients who were over three years of age in order to ensure that they were comparable in age to ICPP cases, clinically did not show rapid height gain, did not have areola hyperpigmentation, did not have bone age more than one year from chronological age, did not show pubertal findings on pelvic ultrasonography and had a dominant FSH response even if LH was above 5 IU/L in GnRH test [18–20].

In addition to the GnRH test, the following criteria were used to make the distinction: growth rate exceeding 6 cm/year, uterine height of more than 35 mm by pelvic ultrasonography (USG), increased corpus/cervix ratio, prominent endometrium thickness, the ovarian volume of 2 ml or more, bone age at the time of presentation is more than one year ahead of the chronological age, loss of in predicted height more than 1 SD compared to the familial target height, bone age change in follow-up being 120% ahead of the chronological age change (1.5–2 years) and skipping pubertal stage in less than three months were accepted as supportive findings of CPP [3, 4, 21, 22]. Patients who developed CPP due to central nervous system pathologies or after premature pubarche were excluded from the study.

The study included patients with Tanner stage 2 and stage 3 breast development. Tanner Stage 4 cases were excluded because comparison with premature thelarche was not possible, and the GnRH response in obese patients was not different from that in normal-weight patients [23].

After administration of intravenous gonadorelin acetate (2.5 μg/kg, maximum 100 μg), FSH, LH, and estradiol levels were measured by electro-chemiluminescence (ECLIA) method in blood samples collected at 0., 20., and 60. minutes.

Growth and BMI charts prepared by Neyzi et al. for Turkish children were used to evaluate anthropometric measurements. BMI values of 85 percentile (≥ 1 SDS) and above were considered overweight, and 95 percentile (≥ 2 SDS) and above were considered obese. Those with BMI-SDS ≥ 1 were divided into the obese-overweight (OW) group and those with BMI-SDS < 1 were divided into the normal weight (NW) group. The patients’ familial target height was calculated and recorded [24].

### Statistical analysis

Descriptive statistical methods (mean, standard deviation, frequency) were used to evaluate the study data. The normality of data distribution was assessed using kurtosis and skewness values and the Kolmogorov–Smirnov test. Student’s t-test and One-Way ANOVA were employed to compare normally distributed groups. Bonferroni post-hoc analysis was used to determine which groups differed. Mann–Whitney U test and Kruskal–Wallis test were used to compare non-normally distributed groups. To determine the diagnostic values of the GnRH test for the ICPP group, receiver operating characteristic (ROC) analysis was performed using the test data from the PT group. A multivariate logistic regression analysis was performed to evaluate the impact of factors such as BMI-SDS, basal hormonal parameters, and pubertal imaging findings on the peak LH cut-off and peak LH/peak FSH ratio. A significance level of *p* < 0.05 was accepted. Data analysis was conducted using the SPSS-23 Statistics program.

## Results

A total of 925 girls with Tanner stage 2 and stage 3 were included in the study. The mean age at diagnosis was 7.9 ± 1.1 years in the ICPP group (*n* = 646, 69.8%) and 6.4 ± 1.4 years in the PT group (*n* = 279, 30.2%).

Among all cases, 455 (49.2%) were classified as overweight (OW). In the PT group, 114 cases (40.9%) had BMI-SDS ≥ 1. Among the NW PT cases, 153 were in stage 2 and 12 in stage 3, while 82 of the OW PT cases were in stage 2 and 32 in stage 3. Of the ICPP patients, 341 (52.8%) had BMI-SDS ≥ 1, 210 and 95 of the NW patients were in stages 2 and 3, and 194 and 147 of the OW patients were in stages 2 and 3, respectively (Table 1).

**Table 1:** Descriptive Characteristics of Premature Telarche and Idiopathic Central Puberty Precocious Cases and Comparison of GnRH Test Results According to Obesity Status

Pubertal Status	PT (*n*=279)			ICPP ( *n*=646)		
BMI-SDS	BMI-SDS < 1 (*n*=165, 59.1%) a_1_	BMI-SDS ≥ 1 (*n*=114, 40.9%) a_2_	*p*	BMI-SDS < 1 (*n*=305, 47.2%) b_1_	BMI-SDS ≥ 1 (*n*:341, 52.8%) b_2_	*p*
Telarch Stage	T2 (*n*=153, 54.8%) T3 (*n*=12, 4.3%)	T2 (*n*=82, 29.4%) T3 (*n*=32, 11.5%)		T2 (*n*=210, 32.5%) T3 (*n*=95, 14.7%)	T2 (*n*=194, 30%) T3 (*n*=147, 22.8%)	
BMI-SDS	−0.17 ±0.81 (−2.75-0.99)	1.69±0.64 (1–3.35)	<0.001	0.57±0.74 (−3.5-0.99)	1.77±0.53 (1–3.1)	*<0.001 **=0.89
Peak FSH (IU/L)	17.57±5.5 (4.03–30.2)	13.74±5.88 (4.2–24.8)	<0.001	11.6±4.12 (3.4–25.8)	10.2±3.8 (4.07–22.1)	*<0.001 **<0.001
Peak LH (IU/L)	4.18±2.26 (0.83–14.4)	3.19±1.73 (0.6–9.99)	**=0.37**	7.3±3.5 (1.4–19.4)	5.29±2.99 (1.1–15.5)	*<0.001 **<0.001
Basal E2 (pg/mL)	6.2±3.44 (2.5–25.5)	6.42±3.07 (4–16)	**=0.9**	14.8±17.1 (0.42–194)	10.6±10.5 (2.5–64)	*<0.001 **<0.001
BA (years)	6.34±1.62 (2.5–9)	7.44±1.73 (2–10.5)	<0.001	9.1±1.6 (3.5–13)	9.6±1.5 (4.2–13)	*<0.001 **<0.001
Δ BA-CA	0.33±0.76	0.46±1.13	**=0.78**	1.16±1.22	1.54±1.23	*<0.001 **<0.001

*ICPP* Idiopathic Central Puberty Precocious, *BMI-SDS* Body Mass Index Standart Deviation Score, *FSH* Follicle Stimulating Hormone, *LH* Luteinising Hormone, *E2* Estradiol, *BA* Bone Age, *CA* Chronological Age, *T2* Thelarche Tanner Stage 2, *T3* Thelarche Tanner Stage 3

* Differentiation between b_1_ and b_2_

** Differentiation between a_2_ and b_2_

In PT cases, the BMI-SDS values in the NW and OW groups were −0.17 ± 0.81 and 1.69 ± 0.64, respectively. In ICPP cases, the BMI-SDS values in the NW and OW groups were 0.57 ± 0.74 and 1.77 ± 0.53, respectively. A significant difference was observed between the NW and OW groups within both PT and ICPP cases. However, the BMI-SDS value of OW PT cases was similar to that of OW ICPP cases (*p* = 0.89) (Table 1).

Mean peak FSH levels were 11.6 ± 4.12 IU/L and 10.2 ± 3.8 IU/L, peak LH levels were 7.3 ± 3.5 IU/L and 5.29 ± 2.99 IU/L, and basal E2 levels were 14.8 ± 17.1 pg/mL and 10.6 ± 10.5 pg/mL in NW and OW ICPP subjects, respectively. These values significantly differed between NW and OW ICPP cases (*p* < 0.001). In NW and OW PT cases, mean peak FSH levels were 17.57 ± 5.5 IU/L and 13.74 ± 5.88 IU/L, peak LH levels were 3.19 ± 1.73 IU/L and 5.29 ± 2.99 IU/L, and basal E2 levels were 6.2 ± 3.44 pg/mL and 6.42 ± 3.07 pg/mL, respectively. In PT cases, peak FSH levels were significantly lower in the OW group compared to the NW group (*p* < 0.001), while no significant differences were observed in peak LH and basal E2 values (*p* = 0.37, *p* = 0.9) (Table 1).

The mean bone age (BA) in the ICPP group was 9.1 ± 1.6 years in NW individuals and 9.6 ± 1.5 years in OW individuals (*p* < 0.001). In the PT group, the mean BA was 6.34 ± 1.62 years in NW individuals and 7.44 ± 1.73 years in OW individuals (*p* < 0.001). Notably, OW PT cases and ICPP cases also differed in this regard. Significant differences were observed in the bone age-chronological age difference (ΔBA-CA) between NW and OW groups in both PT and ICPP cases (*p* < 0.001), except between the NW and OW PT cases (*p* = 0.78). (Table 1).

In a detailed analysis of the ICPP group, divided into overweight and obese subgroups, peak LH levels were significantly lower in the obese subgroup (4.47 ± 2.84 IU/L) compared to the overweight subgroup (5.74 ± 2.3 IU/L) (*p* < 0.001). No significant differences between the two subgroups were observed in peak FSH or basal E2 levels (Table 2).

**Table 2:** Comparison of GnRH Test in ICPP cases according to overweight or obesity status

ICPP			
	BMI-SDS ≥ 1- < 2 (*n* = 224)	BMI-SDS ≥ 2 (*n* = 117)	*p*
Peak FSH (IU/L)	10.43 ± 3.7	9.74 ± 3.93	** = 0.55**
Peak LH (IU/L)	5.74 ± 2.3	4.47 ± 2.84	< 0.001
Basal E2 (pg/mL)	10.85 ± 9.97	10.03 ± 10.46	** = 0.85**

*ICPP* Idiopathic Central Puberty Precocious, *BMI-SDS* Body Mass Index Standart Deviation Score, *FSH* Follicle Stimulating Hormone, *LH* Luteinising Hormone, *E2* Estradiol

In ICPP patients at breast Tanner stage 2 and stage 3, the mean peak LH levels were 5.03 ± 2.78 IU/Land 5.62 ± 3.23 IU/L in the OW group, and 7.21 ± 3.38 IU/L and 7.49 ± 3.88 IU/L in NW group, respectively. In comparing OW ICPP patients, no significant differences were observed in peak FSH, peak LH, or basal E2 levels across breast Tanner stage 2 and stage 3. In contrast, patients in Tanner stage 2 and stage 3 of the NW group differed in these parameters. (Table 3).

**Table 3:** Comparison of GnRH test results according to obesity status and tanner stage with Idiopathic central puberty precocious

ICPP						
	BMI-SDS < 1			BMI-SDS ≥ 1		
	T2 (*n* = 210)	T3 (*n* = 95)	*p*	T2 (*n* = 194)	T3 (*n* = 147)	*p*
Peak FSH (IU/L)	11.77 ± 4.25	11.20 ± 3.82	= 0.001	10.18 ± 3.57	10.21 ± 4.1	** = 0.172**
Peak LH (IU/L)	7.21 ± 3.38	7.49 ± 3.88	= 0.01	5.03 ± 2.78	5.62 ± 3.23	** = 0.97**
Basal E2 (pg/mL)	13.70 ± 12.36	17.42 ± 24.23	< 0.001	10.49 ± 10.3	10.69 ± 9.98	** = 0.95**

*ICPP* Idiopathic Central Puberty Precocious, *BMI-SDS* Body Mass Index Standart Deviation Score, *FSH* Follicle Stimulating Hormone, *LH* Luteinising Hormone, *E2* Estradiol, *T2* Thelarche Tanner Stage 2, *T3* Thelarche Tanner Stage 3

**Fig. 1:**
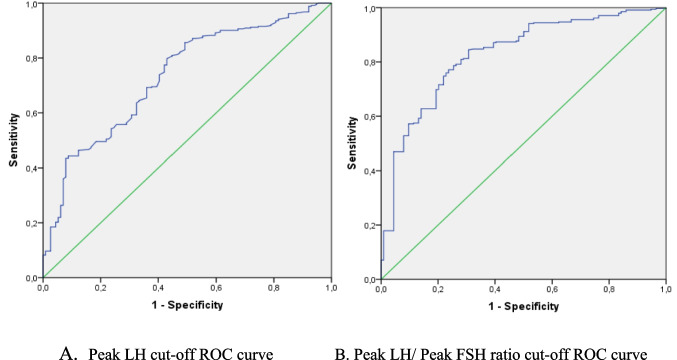
ROC Curves of cut-off value in GnRH test in OW group

**Fig. 2:**
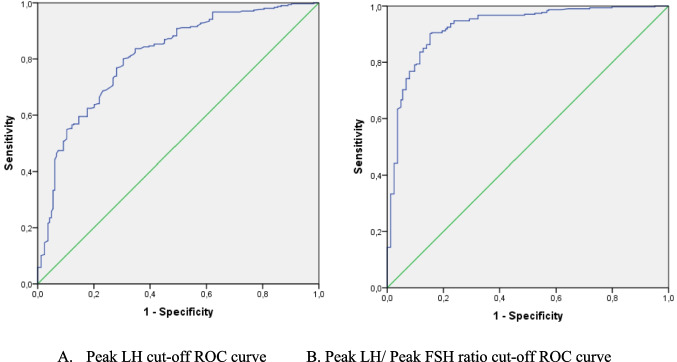
ROC Curves of cut-off value in GnRH test in NW group

Considering the effect of obesity on GnRH test peak LH levels, a different cut-off value was tried to create in OW ICPP patients compared to normal-weight patients. The test results of the ICPP cases were analysed by ROC analysis concerning the results of the PT group, which matched their weight group. In the OW group, the peak LH cut-off was 3.56 IU/L(AUC:0.733) with 69.2% sensitivity and 64% specificity, the peak LH/peak FSH cut-off was 0.29 (AUC:0.828) with 77.1% sensitivity and 76.3% specificity (Fig. 1A, B). In the NW group, the peak LH cut-off value was 4.75 IU/L with 77.1% sensitivity and 70.7% specificity (AUC:0.809), and the peak LH/peak FSH cut-off value was 0.33 (AUC:0.926) with 86.3% sensitivity and 86% specificity (Fig. 2A, B).

To test the values’ robustness, multivariate logistic regression analysis for peak LH cut-off 3.56 IU/L showed that BMI-SDS, basal FSH, and basal E2 significantly affected the value, *p* values < 0.001, < 0.001, and 0.048, respectively (R^2^ = 0.21). In multivariate logistic regression analysis for peak LH/peak FSH ratio cut-off 0.29, BMI-SDS, Basal FSH, Basal E2, BA, and BA-CA difference significantly affected the value, *p* values were 0.003, < 0.001, 0.001, < 0.001 and 0.029, respectively (R^2^ = 0.276) (Table 4).

**Table 4:** Multivariate logistic regression analysis for peak LH cut off model and peak LH/peak FSH cut off model

	Peak LH Cut-off				Peak LH/Peak FSH Ratio			
	Coefficient	OR value	95%Cl	*P* value	Coefficient	OR value	95%Cl	*P* value
BMI-SDS	−0.536	0.585	0.477–0.717	** < 0.001**	−0.296	0.744	0.614–0.902	**0.003**
Basal FSH (IU/L)	0.436	1.547	1.3–1.84	** < 0.001**	0.307	1.36	1.16–1.593	** < 0.001**
Basal LH (IU/L)	−0.137	0.872	0.596–1.277	0.482	−0.090	0.914	0.704–1.185	0.497
Basal E2 (pg/mL)	0.028	1.029	1–1.058	**0.048**	0.064	1.066	1.026–1.108	**0.001**
BA (years)	−0.015	0.985	0.843–1.152	0.854	0.406	1.5	1.288–1.748	** < 0.001**
BA-CA	0.034	1.035	0.836–1.281	0.755	−0.238	0.788	0.636–0.976	**0.029**
Uterus Length	0.015	1.015	0.989–1.041	0.257	0.017	1.017	0.990–1.045	0.218
Over Volume (ml)	0.086	1.090	0.893–1.331	0.389	0.23	1.259	0.965–1.643	0.09

*BMI-SDS* Body Mass Index Standart Deviation Score, *FSH* Follicle Stimulating Hormone, *LH* Luteinising Hormone, *E2* Estradiol, *BA* Bone Age, *CA* Chronological Age, *OR *Odds Ratio, *95%Cl *Confidence Interval

## Discussion

Although obesity is a risk factor for ICPP, it also complicates the diagnosis because LH levels are measured lower [16, 25–27]. This situation requires frequent follow-up and repeat pelvic ultrasound to evaluate fundal/cervical ratio, uterine length, ovarian volume, GnRH tests, and bone age assessment.

Increased leptin levels observed in obesity are thought to play a role in the premature activation of the GnRH pulse generator, which leads to the early onset of pubertal signs [28]. Additionally, increased aromatization in adipose tissue elevates estrogen levels, accelerating bone age [2, 11, 12]. Insulin resistance, a common consequence of obesity, complicates this process by increasing serum levels of ovarian and adrenal steroid hormones while simultaneously reducing SHBG concentrations, and leading to an elevation of free sex steroids that may disrupt the normal regulation of pubertal timing and progression [13, 14, 29, 30]. It should be considered that peak LH response may be lower in obese children due to the suppressive effect of androgens and estrogens on LH, especially in the early pubertal period (Tanner stage 2 and 3) [15, 16, 25, 26].

Sexual dimorphism in the interaction between kisspeptin and GnRH secretion may contribute to the higher prevalence of ICPP in girls [13, 14]. Kisspeptin neurons in the preoptic area of humans mediate the preovulatory gonadotropin surge in females through positive feedback from estrogen, a mechanism absent in males. Animal studies demonstrate that estradiol exerts region-specific effects on Kiss1 expression, it downregulates Kiss1 mRNA in the arcuate nucleus (ARC) but upregulates it in the anteroventral periventricular nucleus (AVPV). Additionally, female rodents exhibit significantly more Kiss1-positive neurons in the AVPV than males, highlighting a potential biological basis for the increased incidence of ICPP in females. [28–31].

McCartney et al.’s study provided valuable insights into the impact of obesity on LH secretion patterns during pubertal maturation in girls. The findings demonstrated that prepubertal and early pubertal obese girls exhibited reduced LH pulse frequency and amplitude, with an absence of nocturnal increases typically observed in their non-obese peers. In contrast, obese girls at later pubertal stages showed elevated LH pulse frequency but reduced amplitude, which was potentially driven by hyperandrogenemia. These alterations highlighted the complex interplay between obesity, LH secretion dynamics, and androgen excess, which could disrupt normal pubertal progression [17]. Another study reported that the sleep-related increase in gonadotropins was blunted in healthy, overweight girls in the early stage of puberty. Still, the peak LH response to a GnRH agonist was unrelated to BMI. They attributed this to using a different test agent (GnRH agonist) and the small number of subjects [32].

Basal LH levels are not always reliable for diagnosing pubertal precocity. Therefore, the GnRH stimulation test is considered the gold standard in all pediatric endocrinology clinics. Studies highlight the negative impact of obesity on peak responses in the GnRH stimulation test. In a study involving 182 girls with CPP, the values for normal-weight versus overweight/obese groups were as follows: basal LH (0.72 ± 0.73 U/L vs. 0.74 ± 0.63 U/L, *p* = 0.870), peak LH (16.37 ± 12.97 U/L vs. 12.53 ± 11.79 U/L, *p* = 0.043), basal FSH (3.14 ± 2.44 U/L vs. 2.99 ± 2.34 U/L, *p* = 0.697), and peak FSH (16.20 ± 8.38 U/L vs. 15.65 ± 7.89 U/L, *p* = 0.658) [33]. Similarly, another study of 234 girls with ICPP found that overweight and obese subjects had significantly lower peak LH (8.95 ± 2.85 U/L vs. 11.97 ± 8.42 U/L), peak FSH (9.60 ± 2.91 U/L vs. 11.17 ± 7.77 U/L), and peak LH/peak FSH ratios (0.99 ± 0.38 vs. 1.24 ± 0.86) compared to normal-weight subjects [27]. Furthermore, in a larger study involving 865 girls, the median peak LH values for normal-weight, overweight, and obese groups were 9.1 IU/L, 8.5 IU/L, and 6.2 IU/L, respectively, while the median peak LH/peak FSH ratios were 0.8, 0.8, and 0.6, respectively [26].

In our clinic, we observed that peak LH levels and peak LH/peak FSH ratios during the GnRH test were lower in overweight and obese girls with ICPP compared to their normal-weight counterparts. For NW ICPP cases, we found mean values 11.6 ± 4.12 IU/L, 7.3 ± 3.5 IU/L, and 14.8 ± 17.1 pg/mL, respectively. OW ICPP patients’ respective values were 10.2 ± 3.8 IU/L, 5.29 ± 2.99 IU/L, and 10.6 ± 10.5 pg/mL. As in other studies, peak FSH and peak LH levels were significantly lower in overweight and obese patients with stage 2 and stage 3 ICPP, indicating that obesity leads to lower measurements of both peak FSH and peak LH in the GnRH test.

Clinical and experimental studies have concluded an independent correlation between peak LH stimulation and BMI-SDS ≥ 1.5 in ICPP girls. This relationship varies across different Tanner stages and may be influenced by adrenal androgens, estradiol, and glucose metabolism [34, 35]. We observed no difference in peak FSH and E2 levels between overweight and obese ICPP patients. In contrast, peak LH levels were significantly lower in obese patients, indicating that as the degree of obesity increases, peak LH values are measured even lower (Table 2). The fact that peak LH levels were lower as the degree of obesity increased, even though E2 levels remained unchanged, suggested mechanisms such as insulin and leptin resistance, decreased SHBG, and elevated androgen levels in obese individuals, independent of sex steroids. In our clinic, dehydroepiandrosterone sülfat and androgen levels are not routinely measured in ICPP cases unless puberty begins with pubarche. Since we cannot assess the suppressive effect of androgens on LH, cases where pubertal development started with pubarche were excluded from the study to avoid this limitation.

A study evaluating the effect of the pubertal stage on peak LH level found that Tanner stage 2 and 3 girls were more sensitive to the negative feedback effect due to relative immaturity in hypothalamic-pituitary function, BMI was not related to peak LH levels, and sensitivity decreased in Tanner stage 4 girls [24]. We conducted our study exclusively on girls in Tanner stages 2 and 3, both to facilitate easier comparison with PT cases and because LH levels during early puberty are more sensitive to the suppressive effect of obesity. When we re-evaluated our ICPP patients as OW and NW according to Tanner stages, we observed that peak FSH, peak LH, and E2 levels were different NW and OW ICPP patients in Tanner stage 2 and stage 3 (Table 3). This data showed us that Stages 2 and 3 hormonal levels were similar in cases with the same obesity status.

Lower threshold values should be considered for diagnosis in GnRH testing in obese girls because a recent study showed that peak serum LH concentrations decreased as BMI-SDS increased in girls with ICPP. In this study, the sensitivity and specificity of peak LH above 4 IU/L for diagnosis in overweight and obese girls with stage 2 and 3 ICPP were found to be 86% and 93%, respectively. It was also reported that serum estradiol levels were not higher in these girls [36].

In our study, peak LH levels in overweight and obese ICPP girls were significantly lower than normal weight. Peak LH cut-off values in the NW and OW groups were established by taking GnRH tests of PT cases as a reference. Peak LH/peak FSH calculation was made considering that peak LH may be above 5 IU/L in PT. In the ICPP OW group, the peak LH cut-off was 3.56 IU/L, and the peak LH/peak FSH was 0.29. In the NW group, the peak LH cut-off was 4.75 IU/L, peak LH/peak FSH was 0.33, and these values were closer to the standard cut-offs for pubertal response to the GnRH test.

Data collected from overweight and obese Tanner stages 2 and 3 ICPP girls evaluated over 12 years showed that their responses to GnRH testing were lower than standard values. It was also observed that as the degree of obesity increased, the peak LH value was measured even lower, independent of the E2 value. In addition, peak LH and peak LH/peak FSH values in normal-weight patients were closer to the standard cutoff values for pubertal response to GnRH testing, supporting the inferred result. The finding of a lower value for peak LH/peak FSH ratio strengthens the study and emphasizes that patients should not be evaluated only with peak LH value. Previous studies have highlighted the negative relationship between BMI and gonadotropin levels, sharing relevant findings on this association [23, 26, 27, 34]. Only one study has proposed a cut-off value for the peak LH level in the GnRH test [36]. In our research, conducted with a large patient cohort, we identified a cut-off value for the peak LH/peak FSH ratio, a critical marker in the assessment and diagnosis of precocious puberty, providing valuable additional insights to complement previous studies. A limitation of the study was that, despite excluding patients with pubarche as the first sign of puberty, adrenal androgen levels were not measured to evaluate their potential suppressive effects on the hypothalamic-pituitary–gonadal axis.

A multivariate logistic regression analysis demonstrated that for a peak LH cut-off of 3.56 variability of BMI-SDS, basal FSH, and basal E2; for a peak LH/peak FSH cut-off of 0.29 variability of BMI-SDS, basal FSH, basal E2, BA, and the BA–CA difference were affecting the values. The R^2^ value of 0.21 for the peak LH model indicates that the predictors included in the model can explain approximately 21% of the variance in peak LH. In this model, BMI-SDS emerged as a significant negative predictor (OR: 0.585, 95% CI: 0.477–0.717, *p* < 0.001), demonstrating a robust inverse association. The peak LH/peak FSH ratio model showed a slightly stronger R^2^ value of 0.276, indicating that the model’s predictors could explain 27.6% of the variance in the ratio. In this model, BMI-SDS was also a significant predictor (OR: 0.744, 95% CI: 0.614–0.902, *p* < 0.001) (Table 4). BMI-SDS was a significant negative predictor in both the peak LH and peak LH/peak FSH models, underlining the inverse relationship between BMI and these pubertal markers. Although the R^2^ values for both models were relatively low, they are considered acceptable given the complex and multifactorial nature of conditions of obesity and puberty.

## Conclusion

Increased adipose tissue in obesity may further complicate the physical examination of glandular breast tissue and potentially mask early signs of puberty. Therefore, bone age assessment, anthropometric measurements, and ultrasound findings in these patients are critical to ensure timely and accurate diagnosis. Notably, diagnostic delays may occur due to inadequate response to GnRH testing causes early menarche, shorter final height compared to the familial target height, and significant psychosocial problems.

GnRH tests performed on overweight and obese girls with ICPP in Tanner stage 2 and 3 revealed values that differed from the standard range. These results underscore the clinical significance of evaluating pubertal hormonal markers in relation to BMI-SDS. However, they also demonstrate the limitations of only utilizing hormonal thresholds for diagnostic purposes.

## Data Availability

Some or all datasets generated during and/or analyzed during the current study are not publicly available.
